# Antibacterial and Anti-Inflammatory Activities of* Physalis Alkekengi* var.* franchetii* and Its Main Constituents

**DOI:** 10.1155/2016/4359394

**Published:** 2016-01-27

**Authors:** Zunpeng Shu, Na Xing, Qiuhong Wang, Xinli Li, Bingqing Xu, Zhenyu Li, Haixue Kuang

**Affiliations:** ^1^Key Laboratory of Chinese Materia Medica (Ministry of Education), Heilongjiang University of Chinese Medicine, No. 28 Heping Road, Xiangfang District, Harbin, Heilongjiang 150040, China; ^2^Departments of Biotechnology, Dalian Medical University, Dalian, Liaoning 116044, China

## Abstract

This study was designed to determine whether the 50% EtOH fraction from AB-8 macroporous resin fractionation of a 70% EtOH extract of* P. Alkekengi* (50-EFP) has antibacterial and/or anti-inflammatory activity both* in vivo* and* in vitro* and to investigate the mechanism of 50-EFP anti-inflammatory activity. Additionally, this study sought to define the chemical composition of 50-EFP. Results indicated that 50-EFP showed significant antibacterial activity* in vitro* and efficacy* in vivo*. Moreover, 50-EFP significantly reduced nitric oxide (NO), prostaglandin E_2_ (PGE_2_), tumor necrosis factor alpha (TNF-*α*), interleukin 1 (IL-1), and interleukin 6 (IL-6) production in lipopolysaccharide- (LPS-) stimulated THP-1 cells. Nitric oxide synthase (iNOS) and cyclooxygenase-2 (COX-2) (examined at the protein level) in THP-1 cells were suppressed by 50-EFP, which inhibited nuclear translocation of p65. Consistent with this anti-inflammatory activity* in vitro*, 50-EFP reduced inflammation in both animal models. Finally, seventeen compounds (8 physalins and 9 flavones) were isolated as major components of 50-EFP. Our data demonstrate that 50-EFP has antibacterial and anti-inflammatory activities both* in vitro* and* in vivo*. The anti-inflammatory effect appears to occur, at least in part, through the inhibition of nuclear translocation of p65. Moreover, physalins and flavones are probably the active components in 50-EFP that exert antibacterial and anti-inflammatory activities.

## 1. Introduction

The continued evolution of dangerous multidrug resistant bacteria has led to significant increases in morbidity and mortality due to bacterial infections. Additionally, many of the currently prescribed antibacterial drugs have significant adverse side effects [1]. Consequently, the urgency for developing new highly effective and safe antibacterials is heightened [2]. To this end, increased attention is being given to the search for antibacterial drugs among natural products and specifically traditional Chinese medicines (TCMs).

One of the most common physiological responses to bacterial infection is the inflammatory response, which is often triggered by changes in humoral and cellular components after tissue injury [3]. The expression of inflammation mediators such as NO, PGE_2_, and cytokines is regulated by the transcriptional regulator NF-*κ*B [4], the expression of which is in turn regulated by a complex signaling cascade [5]. When monocytes are stimulated with LPS, inhibitory kappa B*α* (I*κ*B) is phosphorylated by the I*κ*B kinase *β* (IKK) complex, ubiquitinated, and rapidly degraded, resulting in the release of NF-*κ*B, which can translocate to the nucleus and bind to a host of NF-*κ*B-binding promoter regions. Genes activated by NF-*κ*B included diverse proinflammatory mediators such as iNOS, COX-2, TNF-*α*, IL-1*β*, and IL-6. Moreover, the MAPK signaling pathways stimulated with the LPS-TLR4 combination can activate various transcription factors, such as NF-*κ*B and c-Jun, which also modulate the production of inflammatory mediators and cytokines [6]. In healthy tissues, this inflammatory response plays an essential role in host survival and tissue repair. However, these inflammatory mediators can be overexpressed by some stimuli leading to serious inflammatory disorders [7, 8]. When this occurs, anti-inflammatory drugs are a common therapeutic approach for controlling the inflammatory process. Since bacterial infections can often elicit a problematic inflammatory response, medicines that can provide both an antibacterial and anti-inflammatory response would be of particular therapeutic interest.

*P. alkekengi* (Chinese name: Jindenglong) is a perennial herb taxonomically classified in the Solanaceae family and found widely throughout China.* P. alkekengi* fruits, calyces, roots, and whole plants have been used in traditional Chinese prescriptions, including clinical use of the fruits and calyces. Analysis of ancient medicinal research revealed that* P. alkekengi* has long been used as a traditional Chinese medicine (TCM) for a variety of ailments, including sore throat, cough, eczema, hepatitis, urinary problems, and tumors [9]. In a previous study, our research team identified a* P. alkekengi* extract fraction (50-EFP) effective for treating pharyngitis. Upon further investigation we have determined that the main components of that fraction included physalins and flavones. Because other studies have indicated that physalins and flavones have excellent antibacterial and/or anti-inflammatory activity [10–12], we set out to determine whether 50-EFP also has antibacterial and anti-inflammatory activity.

## 2. Materials and Methods

### 2.1. Plant Material

The calyces of* P. alkekengi* were collected from Maoer Mountain in Heilongjiang province in 2008 and the original plant was identified by Professor Zhenyue Wang of Heilongjiang University of Chinese Medicine. A voucher specimen (Number 20080602) was deposited at the Herbarium of Heilongjiang University of Chinese Medicine, China.

### 2.2. Strains and Reagents

Seven bacterial strains, including four Gram-positive bacteria,* Staphylococcus aureus* (ATCC 26112),* Staphylococcus epidermidis* (ATCC 27342),* Staphylococcus saprophyticus* (ATCC 24582), and* Enterococcus faecium* (ATCC 35667), and three Gram-negative bacteria,* Pseudomonas aeruginosa* (ATCC 27853),* Streptococcus pneumoniae* (NCTC 7465), and* Escherichia coli* (ATCC 87394), were obtained from Beijing ZK Kangtai Biological Co. (Beijing, China). These organisms were stored at −20°C supplemented with 10% glycerol. Beef extract, peptone, and agar powder were purchased from Aoboxing Bio-tech Co. (Beijing China). Roswell Park Memorial Institute (RPMI) 1640, fetal bovine serum (FBS), LPS, penicillin, and streptomycin were obtained from Gibco BRL (NY, USA). Dimethyl sulfoxide (DMSO) was purchased from Beijing Chemical Works (Beijing, China). TNF-*α*, IL-1*β*, IL-6, and PGE2 ELISA detection kit were purchased from R&D Systems (Minneapolis, MN). All antibodies were purchased from Santa Cruz Biotechnology (CA, USA). All chemicals were purchased from Sigma (St. Louis, MO, USA).

### 2.3. Preparation of 50-EFP

The dried calyces of* P. alkekengi* (5 kg) were extracted with 70% ethanol (20 L) under reflux conditions for 2 h, for 2 times, to give a residue (1.4697 kg) after removal of solvent under reduced pressure. Then the extract solution (suspended in H_2_O) flowed slowly through AB-8 macroporous resin chromatographic column (10 × 60 cm) with a flow rate of 2 BV/h. The remaining water extract (300.2 g) was fractioned with H_2_O, 50% (104.6 g, 50-EFP), and 95% EtOH.

### 2.4. Isolation and Identification of Compounds from 50-EFP

The 50-EFP (105.0 g) was subjected to silica gel (200–300 mesh, Qingdao Marine Chemical Co., Qingdao, China) column chromatography with a stepwise CH_2_Cl_2_-MeOH gradient (30 : 1; 20 : 1; 10 : 1; 8 : 1; 5 : 1; 1 : 1, v/v) and finally with MeOH alone, to give eight fractions I–VIII. Fractions of II (19.8 g), III (15.6 g), IV (21.4 g), V (10.3 g), and VI (19.8 g) were further separated by octadecyl silica gel (ODS, 35–55 *μ*m, Fuji) column chromatography with MeOH-H_2_O gradient (10%, 30%, 50%, 70%, and 95%). Subfractions from ODS column chromatography were separated and purified by preparative HPLC (Waters 600) with MeOH-H_2_O to afford 17 compounds (1–17). The structures of compounds 1–17 were determined by detailed NMR (Bruker DPX 400) data analyses, ESI-MS (Waters, Milford, MA, USA), and comparison of their spectral data with literature values.

### 2.5. Chromatographic Conditions

This analysis was performed using a Waters 2695 HPLC system and Symmetry C_18_ column (150 × 4.6 mm, Part Number WATO 45905). Before the analysis, 50-EFP was dissolved in methanol to a concentration of 5.0 mg/mL for the HPLC analysis. The mobile phase consisted of acetonitrile (A) and aqueous phosphoric acid (0.05% v/v) (B). The concentrations of solvent A in the linear gradient program were as follows: 5–8% at 0–15 min, 8–18% at 15–25 min, and 18–35% at 25–60 min. The mobile phase flow rate was 1.0 mL/min and the column temperature was controlled at 35°C. The UV wavelength was 230 nm. Ten microliters of samples was injected to the column.

### 2.6. Animals

Male ICR mice (6–8 weeks) were used throughout the experiments. The animals were housed under standard laboratory conditions (temperature at 25 ± 1°C, humidity at 60%, and light from 6 a.m. to 6 p.m.), given standard rodent chow, and allowed free access to water. All procedures were approved by the Animal Care and Use Committee of China Pharmaceutical University and conform to the revised Guide for the Care and Use of Laboratory Animals published by the US National Institute of Health (NIH) Publication Numbers 85-23 (1996).

### 2.7. Acute Toxicity

The acute toxicity test for 50-EFP evaluated any possible toxicity. ICR mice (*n* = 10 in each) were tested by orally administering different doses of 50-EFP by increasing or decreasing the dose according to the responses of animals [13]. The given maximum dose was 12.8 g/kg, while the control group only received distilled water. All animals were observed for any gross effect or mortality within 24 h.

### 2.8. Antibacterial Activity

The antibacterial activities of 50-EFP were evaluated by determining the minimum inhibitory concentration (MIC) and minimal bactericidal concentration (MBC)* in vitro*. The MIC of 50-EFP for the isolated bacterial strains were determined by tube dilution method as previously described with as light modification [14]. Briefly, bacterial strains were grown on Mueller-Hinton (MH) agar plates and suspended in MH broth. The inoculum suspensions were prepared from 6 h broth cultures incubation and adjusted to obtain a 0.5 McFarland standard turbidity and were then diluted 1000-fold with the respective medium to the concentration of 1.5 × 10^5^ CFU/mL. Twofold serial diluted concentrations of 50-EFP were added in MH broth ranging from 0.20625 to 26.4 mg/mL. To adjust the interference by plant pigments, a parallel series of mixtures with uninoculated broth was prepared. The bacterial suspensions were aerobically incubated for 18 h at 37°C. Triplicate samples were performed for each test concentration. MIC was defined as the lowest concentration inhibiting visible growth.

The MBC determination was carried out by spreading 0.1 mL of the cultures in each tube without visible growth onto sample free MH agar and incubated for 18 h. MBC was considered as the highest dilution at which almost bacterial inoculum was killed. The experiments were performed in triplicate.

### 2.9. *Pseudomonas aeruginosa* or* Staphylococcus aureus*-Induced Sepsis

Mice were randomly divided into six groups to receive 0.9% saline (normal and saline group, i.p.), amoxicillin (200 mg/kg), or 50-EFP (160, 320, and 640 mg/kg, i.p.). The mice were challenged intraperitoneally with* Pseudomonas aeruginosa* or* Staphylococcus aureus* (0.5 mL) containing 1.5 × 10^5^ CFU/mL to induce sepsis model, respectively. Mice were treated with 50-EFP and amoxicillin, respectively, for one day (1, 6, and 12 h) before infection and 1, 6, and 12 h after infection. The mortality of the mice was observed for 24 h.

### 2.10. Cell Culture

The THP-1 cell, obtained from Cell Bank of the Chinese Academy of Sciences (Shanghai, China), was cultured in RPMI 1640 containing 10% heat-inactivated FBS supplemented with 1% penicillin/streptomycin under standard conditions. The cells were kept at 37°C in a humidified atmosphere of 5% CO_2_. The cells were seeded in 96-well (1 × 10^5^ cells/mL) or 6-well (1 × 10^6^ cells/mL) plates.

### 2.11. Cell Viability Assay

Cell viability was assessed by morphology and by reduction of MTT by mitochondrial dehydrogenases, according to the manufacturer's instruction (Sigma). THP-1 cells were treated with 50-EFP (0.2, 1, 5, 25, 100, and 500 *μ*g/mL) and the plates were incubated for 24 or 48 h. The cells were then washed once before adding 100 *μ*L of FBS-free medium containing MTT (5 mg/mL). After 4 hours of incubation at 37°C, the medium was discarded and the formazan blue that formed in the cells was dissolved in dimethyl sulfoxide (DMSO). The optical density was measured at 570 nm.

### 2.12. NO Quantification

The accumulation of NO, a stable end product extensively used as an indicator of NO production, was assayed using the Griess reagent. THP-1 cells were seeded on 6-well tissue culture plates at 1 × 10^6^ cells/mL containing the medium (2 mL), after which they were incubated with LPS (1 *μ*g/mL) for 24 h. 50-EFP (25, 50, and 100 *μ*g/mL) was pretreated for 6 h before LPS stimulation. The supernatants were mixed with equal amounts of Griess reagent. Samples were incubated at room temperature for 10 min. The absorbance was subsequently read at 540 nm using a microplate reader.

### 2.13. Determining TNF-*α*, IL-1*β*, IL-6, and PGE_2_ Production

The amount of proinflammatory cytokines released in the culture medium was measured using TNF-*α*, IL-1*β*, IL-6, and PGE_2_ ELISA kits based on the quantitative sandwich enzyme immunosorbent technique. THP-1 cells were cultured in six-well plates. 50-EFP (25, 50, and 100 *μ*g/mL) was pretreated for 6 h before LPS stimulation. After treatment, THP-1 cells were incubated with LPS (1 *μ*g/mL) for 24 h. Levels of TNF-*α*, IL-1*β*, IL-6, and PGE_2_ in the culture media were quantified using ELISA detection kits. The absorbance was read at a wavelength of 450 nm using a microplate reader.

### 2.14. Nuclear Extract Protein Preparation

After treatment with 50-EFP, THP-1 cells were harvested, washed with PBS, centrifuged, and resuspended in ice-cold buffer A (10 mM HEPES (pH 7.0), 1.5 mM MgCl_2_, 10 mM KCl, 0.5 mM DTT, and 0.2 mM PMSF). After 10 min of ice incubation, the cells were again centrifuged, resuspended in buffer C (20 mM HEPES (pH 7.9), 20% glycerol, 420 mM NaCl, 1.5 mM MgCl_2_, 0.2 mM EDTA, 0.5 mM DTT, and 0.2 mM PMSF), and incubated for 20 min at 0°C. After vortex mixing, the resulting suspension was centrifuged, and the supernatant (nuclear extract) was stored at −70°C. The protein concentration of the nuclear extract was determined by the Bradford method using the Bio-Rad protein assay kit (Bio-Rad Laboratories, Hercules, CA, USA).

### 2.15. Western Blot Analysis

THP-1 cells in serum-free RPMI 1640 medium were incubated with 50-EFP for 6 h before LPS treatment. After treatment, cells were harvested, washed with PBS, and lysed with cell lysis buffer containing 1% phenylmethylsulfonyl fluoride. The lysate was centrifuged for 15 min at 12000 ×g and 4°C to remove insoluble materials. Supernates were then collected. Protein concentration was measured by bicinchoninic acid assay. Equal amounts of protein (20 *μ*g) from each sample were separated by 10% SDS-PAGE and transferred onto a nitrocellulose membrane (Millipore Corporation, USA). Nonspecific sites were blocked by the incubating membranes (2 h at room temperature) in 5% (w/v) nonfat milk powder in Tris-buffered saline containing 0.05% (v/v) Tween-20 (TBS-T). Thereafter, the membranes were incubated overnight at 4°C with primary antibodies from Santa Cruz Biotechnology. The membranes were washed with TBS-T and incubated with the appropriate secondary HRP-conjugated antibodies at 1 : 1000 dilution. Following a 30 min wash, the membranes were visualized by enhanced chemiluminescence. Band intensity was measured and quantified.

### 2.16. Xylene-Induced Ear Edema and Cotton Pellet Implantation in Mice

The xylene-induced ear edema test was used to assess anti-inflammatory activity following the procedure described previously. ICR mice were randomized into five groups (*n* = 10), including a control group, a positive group (aspirin-treated, 80 mg/kg, i.g.), and 50-EFP treatment groups (50, 100, and 200 mg/kg, i.p.). Test groups of mice were given 50-EFP once every day for 3 consecutive days. Xylene (0.05 mL) was applied to the anterior and posterior surfaces of the right ear of each mouse 1 h after the last administration of 50-EFP. The left ear remained untreated and saved as a control. Ear disk of 7.0 mm in diameter was punched out and weighed. The weight difference between the left and the right ear disk of the same animal was evaluated as the extent of edema.

Two cotton pellets, weighing 10 ± 1 mg each, sterilized in a hot air oven at 120°C for 2 h, were implanted subcutaneously through a skin incision, one on each side of the abdomen of the animal under light ether anesthesia and sterile technique [15]. Control groups received the vehicle (saline, 10 mL/kg), while the positive group was treated with 200 mg/kg of Qingkailing particles. Test groups of mice were given 50-EFP (50, 100, and 200 mg/kg, i.p.) once per day for 7 consecutive days simultaneously. On the 8th day after implantation, mice were anesthetized with pentobarbital sodium (50 mg/kg, i.p.). Both implanted cotton pellets were dissected and dried at 60°C for 18 h, and their dry weight was calculated.

### 2.17. Statistical Analysis

Data from at least three independent experiments were expressed as mean ± SD. Statistical comparisons between different groups were performed using one-way ANOVA, followed by Student-Newman-Keuls test. The level of significance was set at *p* < 0.05.

## 3. Results

### 3.1. Compounds from 50-EFP

Seventeen known compounds (1–17) were isolated from 50-EFP. Eight physalins (1–8) were identified as physalin P (1), physalin G (2), physalin O (3), physalin N (4), physalin B (5), physalin E (6), physalin J (7), and physalin F (8), and nine flavones (9–17) were identified as apigenin-7-O-*β*-D-glucoside (9), apigenin-7, 4′O-*β*-D-di-glucoside (10), luteolin (11), chrysoeriol-7-O-*β*-D-glucoside (12), diosmetin-7-O-*β*-D-glucoside (13), luteolin-4′-O-*β*-D-glucoside (14), luteolin-3′-O-*β*-D-glucoside (15), luteolin-7-O-*β*-D-glucoside (16), and luteolin-7,3′-O-*β*-D-di-glucoside (17) by comparing their NMR spectroscopic and ESI-MS data with the literature values, respectively.

### 3.2. Acute Toxicity of 50-EFP in Mice

An acute toxicity study in mice indicated that the administration of graded doses of 50-EFP up to 12.8 g/kg produced no adverse effects on the general behavior or appearance of the mice and all the mice survived through the experimental evaluation period.

### 3.3. Antibacterial Activity

#### 3.3.1. Antibacterial Activity* In Vitro*

The antibacterial activity of 50-EFP was quantitatively assessed by determining the MIC and MBC against seven bacterial strains, including four Gram-positive bacteria and three Gram-negative bacteria (Table 1). The results showed that the 50-EFP possessed antibacterial potential against each of the tested strains with MIC and MBC values ranging from 0.825 to 1.65 and 1.65 to 13.20 mg/mL, respectively.

#### 3.3.2. Antibacterial Activity* In Vivo*

A* Staphylococcus aureus-* or* Pseudomonas aeruginosa*-induced sepsis model in mice was used to investigate the antibacterial efficacy of 50-EFP* in vivo* (Table 2). The mortality rate for untreated control groups infected with either* S. aureus* or* P. aeruginosa* was 100% within 24 h. At doses of 160, 320, and 640 mg/kg, 50-EFP significantly reduced mortality rates to 33.3%, 33.3%, and 58.3%, respectively, in* S. aureus*-infected mice. Likewise, at doses 160, 320, and 640 mg/kg, 50-EFP reduced mortality rates to 50%, 58.3%, and 58.3%, respectively, in* P. aeruginosa*-infected mice.

### 3.4. *In Vitro* Anti-Inflammatory Activity of 50-EFP

**Figure 1:**
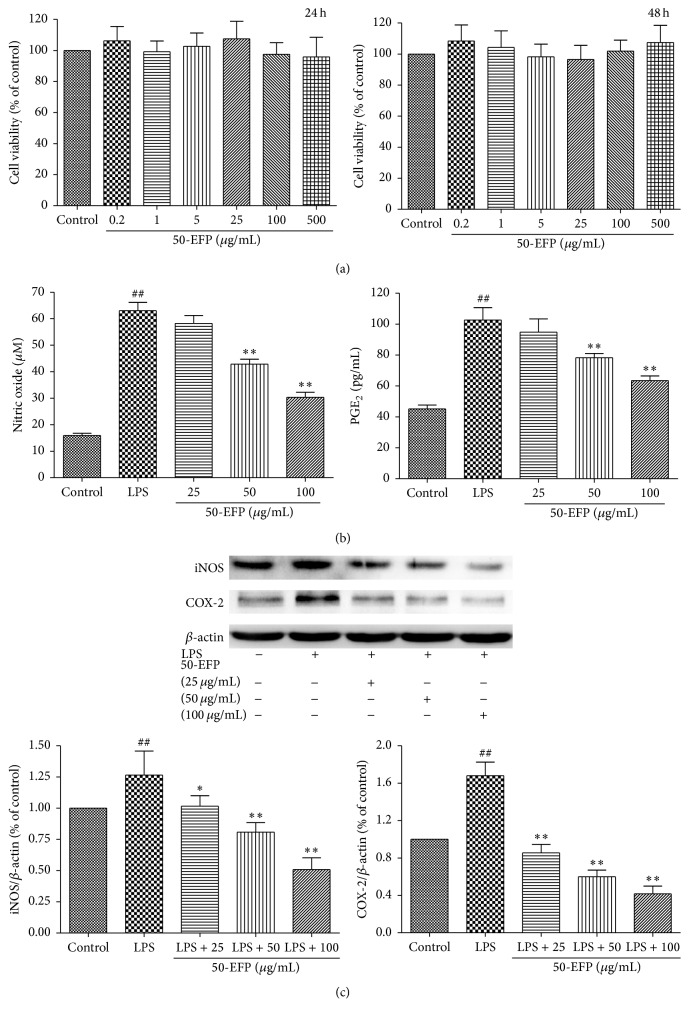
Effects of 50-EFP on THP-1 cell viability. (a) Cells were incubated with increasing concentrations of 50-EFP (0.2, 1, 5, 25, 100, and 500 *μ*g/mL) for 12 h or 24 h. Cell viability was measured by the MTT assay. (b) Effect of 50-EFP on LPS-induced NO and PGE_2_ production in THP-1 cells. THP-1 cells were pretreated with 50-EFP for 6 h before being incubated with LPS for 24 hours. The culture supernatant was analyzed for NO or PGE_2_ production. (c) Total cellular protein was isolated and LPS-induced iNOS and COX-2 expression levels were measured using Western blotting analysis. ^##^ *p* < 0.01 versus control, ^*∗*^ *p* < 0.05; ^*∗∗*^ *p* < 0.01 versus LPS only group. Data are representative of three independent experiments.

The anti-inflammatory effect of 50-EFP was assessed in LPS-stimulated THP-1 cells, a human monocytic cell line. First, the potential cytotoxicity of 50-EFT toward THP-1 cells was assessed in a standard viability assay using the colorimetric dye 3-(4,5-dimethylthiazol-2-yl)-2,5-diphenyltetrazolium bromide (MTT). Viability was unaffected by 50-EFT concentrations up to 500 *μ*g/mL (Figure 1(a)). Next, we investigated the 50-EFP effects on suppressing the levels of NO and PGE_2_ in the LPS-stimulated promonocytic cells. Pretreatment with 50-EFP prior to LPS stimulation caused a significant reduction in NO and PGE_2_ production in a 50-EFP concentration-dependent manner (*p* < 0.01; 50 and 100 *μ*g/mL, Figure 1(b)). Additionally, Western blot analysis indicated that the expression of the genes that synthesize NO and PGE_2_ (iNOS and COX-2, resp.) was markedly reduced in a dose-dependent fashion by 50-EFP pretreatment (Figure 1(c)). Notably, 50-EFP pretreatment did not affect the expression level of the *β*-actin control. These results suggest that 50-EFP suppresses both iNOS and COX-2 expression at the transcriptional level in addition to the protein level, resulting in the reduced expression of NO and PGE_2_.

**Figure 2:**
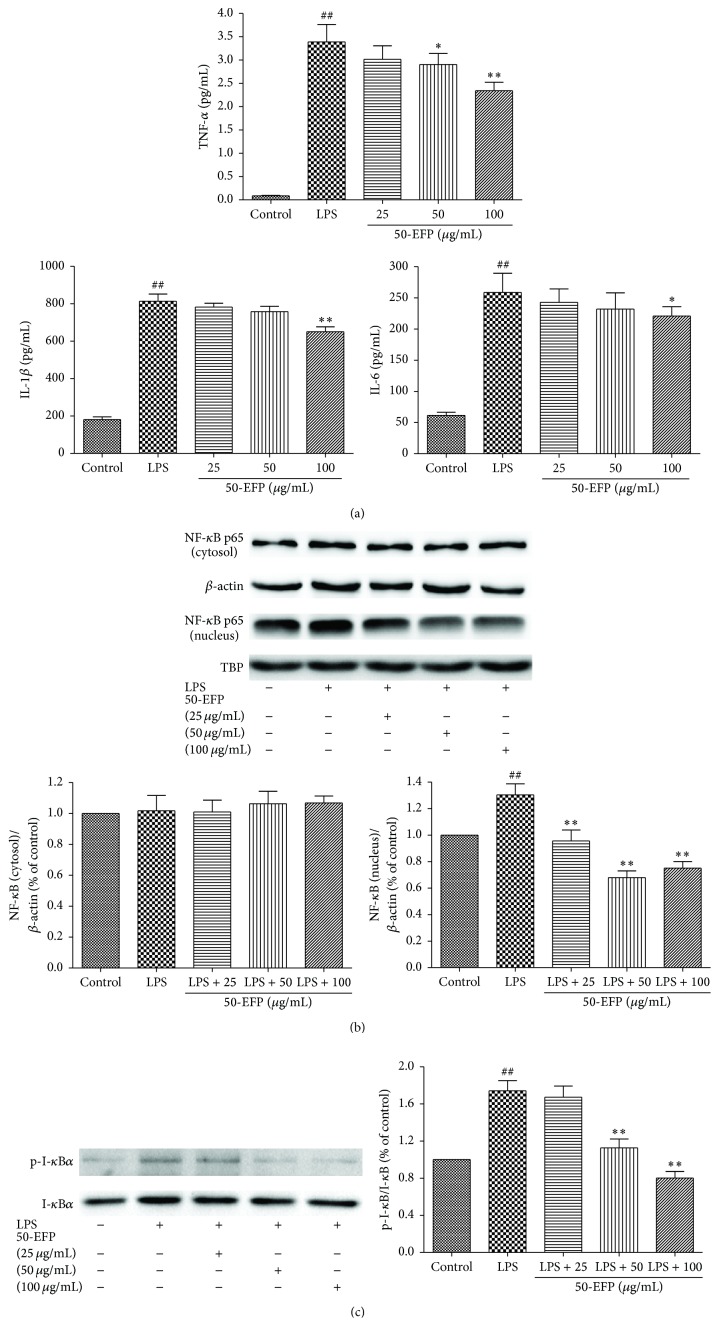
Effect of 50-EFP on LPS-induced TNF-*α*, IL-1*β*, and IL-6 cytokine production. (a) THP-1 cells were pretreated with 50-EFP for 6 h before being incubated with LPS for 24 hours. Production of TNF-*α*, IL-1*β*, and IL-6 cytokine was measured by ELISA. (b, c) Effect of 50-EFP on translocation of the NF-*κ*B (p65) subunit into the nucleus and release of I-*κ*B*α* into the cytosol upon LPS stimulation. The cells were treated with LPS alone or with LPS and 50-EFP for 6 hours. The level of I-*κ*B*α* protein in the cytosol and NF-*κ*B (p65) protein present in the cytosol and nucleus was determined by the Western blot analysis using anti-I-*κ*B*α* or anti-NF-*κ*B (p65) antibody. *β*-actin and TBP were used for cytosolic and nuclear control protein, respectively (in relative protein density units). ^##^ *p* < 0.01 versus control, ^*∗*^ *p* < 0.05; ^*∗∗*^ *p* < 0.01 versus LPS only group. Data are representative of three independent experiments.

The expression of iNOS and COX-2 is normally induced by NF-*κ*B activation. For this to occur the p65 protein NF-*κ*B must translocate from the cytosol to the nucleus—a process that is mediated by phosphorylation and degradation of the I-*κ*B*α* subunit. Therefore, we assessed whether the impact of 50-EFP on iNOS and COX-2 expression occurs via an effect on p65 translocation by Western blot analysis (Figures 2(b) and 2(c)). Interestingly, 50-EFP pretreatment does repress LPS-induced nuclear translocation of p65 and I-*κ*B*α* phosphorylation almost completely, implying that 50-EFP prevents I-*κ*B*α* degradation and NF-*κ*B activation. Next, ELISA analysis was used to investigate the effect of 50-EFP on the LPS-stimulated expression of other NF-*κ*B-regulated genes: TNF-*α*, IL-1*β*, and IL-6. Whereas LPS stimulation significantly increased the production of the cytokines in untreated cells, 50-EFP pretreatment at 25, 50, and 100 *μ*g/mL significantly suppressed the release of TNF-*α*, IL-1*β*, and IL-6 in a dose-dependent manner (Figure 2(a)).

### 3.5. Inhibition of Acute Ear Edema Induced by Xylene and Cotton Pellet-Induced Granulomatous Tissue Formation

**Figure 3:**
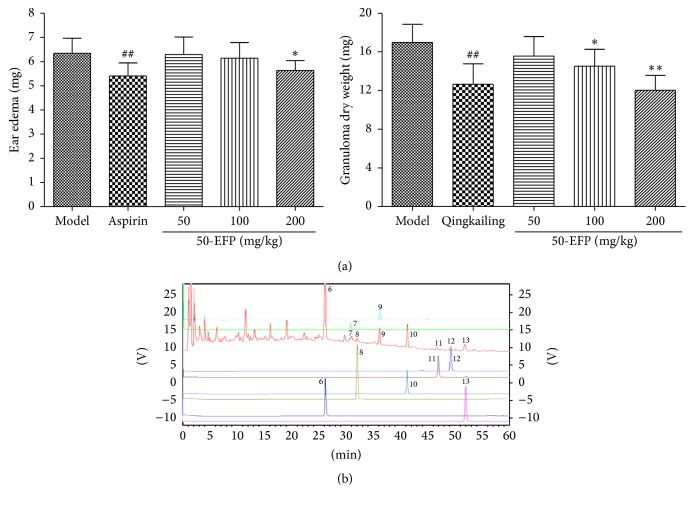
Anti-inflammatory effects of 50-EFP in mice. (a) Xylene-induced ear edema in mice and cotton pellet granuloma in mice. Data are presented as mean ± SD, *n* = 10. ^*∗*^ *p* < 0.05, ^*∗∗*^ *p* < 0.01, as compared with the model group. (b) HPLC chromatograms of 50-EFP, luteolin-7-O-*β*-D-glucoside (6), apigenin-7-O-*β*-D-glucoside (7), diosmetin-7-O-*β*-D-glucoside (8), physalin J (9), physalin F (10), physalin O (11), physalin B (12), and physalin P (13).

A xylene-induced acute ear edema model in mice was used to investigate whether 50-EFP exerts anti-inflammatory effects* in vivo*. Whereas untreated animals show significant ear edema, treatment with 200 mg/kg 50-EFP significantly reduced ear edema (Figure 3(a)). These results indicate that 50-EFP does repress the inflammatory response induced by xylene. Additionally, 50-EFP significantly inhibited granulomatous tissue formation induced by cotton pellet in a dose-dependent manner (*p* < 0.05, 100 mg/kg; *p* < 0.01, 200 mg/kg).

### 3.6. HPLC Analysis of 50-EFP

The main components profile of 50-EFP was analyzed via HPLC. The representative chromatogram is shown in Figure 3(b). The identification of constituent of 50-EFP was based on the retention times and UV spectrum in comparison with authentic standards at a wavelength of 230 nm. Peak purity check and identification were conducted via a 210–400 nm UV scan through a diode array detector. Eight components (luteolin-7-O-*β*-D-glucoside (6), apigenin-7-O-*β*-D-glucoside (7), diosmetin-7-O-*β*-D-glucoside (8), physalin J (9), physalin F (10), physalin O (11), physalin B (12), and physalin P (13)) were identified in 50-EFP.

## 4. Discussion

*Physalis alkekengi* var.* franchetii* (Solanaceae) is a herb widely used in popular medicine for its antifebrile and detoxification effects and to treat sore throats. In a previous study, we found that 50-EFP can also be used to treat pharyngitis. Pathologies causing pharyngitis include a wide range of conditions with two primary underlying causes: inflammation and bacterial infection of pharyngeal mucosa [16–18]. Therefore, we investigated whether a key fraction of* P. alkekengi* extract known as 50-EFP has inherent antibacterial and anti-inflammatory activities.

Despite the advances in antibiotics in the past 70 years, infectious diseases still are an important cause of worldwide morbidity and mortality and account for approximately one-half of all deaths in tropical countries [19, 20]. Therefore, new antibacterial drugs with novel targets are necessary. The antibacterial activity of 50-EFP was assessed by broth dilution method* in vitro*. Bacterial infection was observed in both Gram-positive and Gram-negative strains. In the present study, we found that 50-EFP not only inhibited the growth of both strains (MIC), but also killed them (MBC)* in vitro*. In addition, we also assessed the antibacterial activity of 50-EFP toward* S. aureus* and* P. Aeruginosa in vivo*.* P. aeruginosa* is the most common pathogen that causes respiratory pneumonia, gastrointestinal disorders, bacteremia, and skin infections [21] and* S. aureus* is a major nosocomial pathogen that causes serious infections such as toxic shock syndrome and necrotizing pneumonia [22, 23]. We found that this antibacterial effect was also observed in an animal model of septic infection by either* S. aureus* or* P. aeruginosa*. These results confirm that 50-EFP does have broad-spectrum antibacterial activity and the potential for development as a systemic antibiotic.

In addition to its antibacterial activity, 50-EFP also has significant anti-inflammatory properties. In LPS-stimulated monocytes, 50-EFP inhibits the production and expression of a number of cytokines and inflammatory mediators, including NO, PGE_2_, iNOS, COX-2, TNF-*α*, IL-1*β*, and IL-6, and the effect appears to be dependent on 50-EFP concentration. The basis of the 50-EFP anti-inflammatory activity appears to be inhibition of NF-*κ*B activation and nuclear translocation. Activation of NF-*κ*B requires phosphorylation and degradation of an NF-*κ*B repressor, I-*κ*B*α* [24, 25]. Once freed from I-*κ*B*α*, NF-*κ*B can translocate to the nucleus, where it is an inducer of a number of genes, including the inflammatory response genes repressed by 50-EFP [6]. Our results reveal that treatment with 50-EFP significantly represses I-*κ*B*α* phosphorylation and NF-*κ*B nuclear translocation, and consistent with this, we found that the expression of other NF-*κ*B-regulated genes is also repressed by 50-EFP.

Importantly, the* in vitro* anti-inflammatory activity of 50-EFP is also manifested* in vivo*. In both an ear edema model and a cotton pellet granulomatous tissue model, 50-EFP showed a profound ability to reduce acute and chronic inflammatory reactions. These data provide a better understanding of the positive impact of* P. alkekengi* var.* franchetii* (Solanaceae) treatment of pharyngitis and indicate significant potential for the continued development of combined antibacterial/anti-inflammatory therapies based on the 50-EFP extract.

To further develop the potential therapies of 50-EFP, we had isolated and identified its components. We found that 50-EFP contains eight major components in two key medical classes: physalins and flavones. Previous studies have reported that physalins have suppressive activities on macrophage and lymphocyte cultures* in vitro* and also inhibit the production of proinflammatory mediators such as TNF-*α* [12]. Similarly, flavones have been reported to show significant antibacterial activity via the diffusion method and can suppress chemokine production in human monocyte THP-1 cells [10]. Thus, it is highly likely that the beneficial effects of 50-EFP, and thus* P. alkekengi*, are due to a unique combination of specific physalins and flavones found in the 50-EFP extract. Our mechanistic analysis and component identification should therefore provide a solid basis for more advanced evaluation and development of these components as potential therapeutic agents. This study also confirms the potential and underlying basis for treating inflammation-causing bacterial infections with traditional oriental medicines.

## Figures and Tables

**Table 1:** MIC and MBC of 50-EFP.

Strains	50-EFP (mg/mL)	Ceftriaxone sodium (*μ*g/mL)
*Staphylococcus aureus *ATCC 26112 (MIC)	0.825	8.00
*Staphylococcus aureus *ATCC 26112 (MBC)	>3.30	16.00
*Staphylococcus epidermidis* ATCC 27342 (MIC)	0.825	8.00
*Staphylococcus epidermidis *ATCC 27342 (MBC)	6.60	16.00
*Staphylococcus saprophyticus* ATCC 24582 (MIC)	1.65	8.00
*Staphylococcus saprophyticus* ATCC 24582 (MBC)	3.30	16.00
*Enterococcus faecium* ATCC 35667 (MIC)	1.65	4.00
*Enterococcus faecium* ATCC 35667 (MBC)	>6.60	8.00
*Pseudomonas aeruginosa* ATCC 27853 (MIC)	0.825	0.06
*Pseudomonas aeruginosa* ATCC 27853 (MBC)	1.65	8.00
*Streptococcus pneumoniae *NCTC 7465 (MIC)	0.825	0.06
*Streptococcus pneumoniae *NCTC 7465 (MBC)	>13.20	8.00
*Escherichia coli *ATCC 87394 (MIC)	1.65	8.00
*Escherichia coli *ATCC 87394 (MBC)	3.30	16.00

**Table 2:** Protective effect of 50-EFP in *Staphylococcus aureus* and *Pseudomonas aeruginosa* infection-induced sepsis in mice.

Groups	Number	*Staphylococcus aureus* (24 h)		*Pseudomonas aeruginosa* (24 h)	
		Mortality	Mortality rate (%)	Mortality	Mortality rate (%)
Model	12	12	100	12	100
Amoxicillin (200 mg/kg)	12	2	16.7	3	25.0
50-EFP (640 mg/kg)	12	4	33.3	6	50.0
50-EFP (320 mg/kg)	12	4	33.3	7	58.3
50-EFP (160 mg/kg)	12	7	58.3	7	58.3

## References

[1] Covington R. T. (1988). Management of diarrhea. *Fact and Comparison Drug News Letter*.

[2] Mutai C., Bii C., Vagias C., Abatis D., Roussis V. (2009). Antimicrobial activity of *Acacia mellifera* extracts and lupane triterpenes. *Journal of Ethnopharmacology*.

[3] de Cruvinel W. M., Mesquita D., Araújo J. A. P. (2010). Sistema imunitário—parte I. Fundamentos da imunidade inata com ênfase nos mecanismos molecuares e celulares da resposta inflamatória. *Revista Brasileira de Reumatologia*.

[4] Makarov S. S. (2000). NF-*κ*b as a therapeutic target in chronic inflammation: recent advances. *Molecular Medicine Today*.

[5] Becker S., Mundandhara S., Devlin R. B., Madden M. (2005). Regulation of cytokine production in human alveolar macrophages and airway epithelial cells in response to ambient air pollution particles: further mechanistic studies. *Toxicology and Applied Pharmacology*.

[6] Ruland J., Mak T. W. (2003). Transducing signals from antigen receptors to nuclear factor *κ*B. *Immunological Reviews*.

[7] Pierce G. F. (1990). Macrophages: important physiologic and pathologic sources of polypeptide growth factors. *American Journal of Respiratory Cell and Molecular Biology*.

[8] Simons R. K., Junger W. G., Loomis W. H., Hoyt D. B. (1996). Acute lung injury in endotoxemic rats is associated with sustained circulating IL-6 levels and intrapulmonary cinc activity and neutrophil recruitment—role of circulating TNF-*α* and IL-1*β*. *Shock*.

[9] Qiu L., Zhao F., Jiang Z.-H. (2008). Steroids and flavonoids from *Physalis alkekengi* var. *franchetii* and their inhibitory effects on nitric oxide production. *Journal of Natural Products*.

[10] Kosalec I., Pepeljnjak S., Bakmaz M., Vladimir-Knežević S. (2005). Flavonoid analysis and antimicrobial activity of commercially available propolis products. *Acta Pharmaceutica*.

[11] Huang C.-H., Kuo P.-L., Hsu Y.-L. (2010). The natural flavonoid apigenin suppresses Th1- and Th2-related chemokine production by human monocyte THP-1 cells through mitogen-activated protein kinase pathways. *Journal of Medicinal Food*.

[12] Pinto N. B., Morais T. C., Carvalho K. M. B. (2010). Topical anti-inflammatory potential of Physalin E from *Physalis angulata* on experimental dermatitis in mice. *Phytomedicine*.

[13] Roux S., Sablé E., Porsolt R. D. (2005). Primary observation (Irwin) test in rodents for assessing acute toxicity of a test agent and its effects on behavior and physiological function. *Current Protocols in Toxicology*.

[14] Ogata M., Hoshi M., Urano S., Endo T. (2000). Antioxidant activity of eugenol and related monomeric and dimeric compounds. *Chemical and Pharmaceutical Bulletin*.

[15] Swingle K. F., Shideman F. E. (1972). Phases of the inflammatory response to subcutaneous implantation of a cotton pellet and their modification by certain anti-inflammatory agents. *The Journal of Pharmacology and Experimental Therapeutics*.

[16] Sun Y., Zang Z., Xu X. (2011). Experimental investigation of the immunoregulatory and anti-inflammatory effects of the traditional Chinese medicine ‘li-Yan Zhi-Ke Granule’ for relieving chronic pharyngitis in rats. *Molecular Biology Reports*.

[17] Lopardo G., Yahni D. (2013). On the role of groups C and G streptococci in acute pharyngitis. *Medicina*.

[18] Lopardo H. A. (2013). Groups C and G streptococcal pharyngitis. *Medicina*.

[19] Tekwu E. M., Pieme A. C., Beng V. P. (2012). Investigations of antimicrobial activity of some Cameroonian medicinal plant extracts against bacteria and yeast with gastrointestinal relevance. *Journal of Ethnopharmacology*.

[20] Moellering R. C., Graybill J. R., McGowan J. E., Corey L. (2007). Antimicrobial resistance prevention initiative-an update: proceedings of an expert panel on resistance. *The American Journal of Medicine*.

[21] Silva J. C., Rodrigues S., Feás X., Estevinho L. M. (2012). Antimicrobial activity, phenolic profile and role in the inflammation of propolis. *Food and Chemical Toxicology*.

[22] Boyle-Vavra S., Daum R. S. (2007). Community-acquired methicillin-resistant Staphylococcus aureus: the role of Panton-Valentine leukocidin. *Laboratory Investigation*.

[23] Dinges M. M., Orwin P. M., Schlievert P. M. (2000). Exotoxins of *Staphylococcus aureus*. *Clinical Microbiology Reviews*.

[24] An H.-J., Kim I.-T., Park H.-J., Kim H.-M., Choi J.-H., Lee K.-T. (2011). Tormentic acid, a triterpenoid saponin, isolated from *Rosa rugosa*, inhibited LPS-induced iNOS, COX-2, and TNF-*α* expression through inactivation of the nuclear factor-*κ*b pathway in RAW 264.7 macrophages. *International Immunopharmacology*.

[25] Perkins N. D. (2007). Integrating cell-signalling pathways with NF-*κ*B and IKK function. *Nature Reviews Molecular Cell Biology*.

